# Metabolomic Analysis Reveals That the Moor Frog *Rana arvalis* Uses Both Glucose and Glycerol as Cryoprotectants

**DOI:** 10.3390/ani12101286

**Published:** 2022-05-17

**Authors:** Sergei V. Shekhovtsov, Nina A. Bulakhova, Yuri P. Tsentalovich, Ekaterina A. Zelentsova, Ekaterina N. Meshcheryakova, Tatiana V. Poluboyarova, Daniil I. Berman

**Affiliations:** 1 Institute of the Biological Problems of the North FEB RAS, 685000 Magadan, Russia; sigma44@mail.ru (N.A.B.); katusha@ibpn.ru (E.N.M.); dberman@mail.ru (D.I.B.); 2 Institute of Cytology and Genetics SB RAS, 630090 Novosibirsk, Russia; tanita11@mail.ru; 3 International Tomography Center SB RAS, 630090 Novosibirsk, Russia; yura@tomo.nsc.ru (Y.P.T.); zelentsova@tomo.nsc.ru (E.A.Z.); 4 Department of Chemical and Biological Physics, Novosibirsk State University, 630090 Novosibirsk, Russia

**Keywords:** freezing tolerance, cryoprotectants, metabolomics analysis, *Rana arvalis*

## Abstract

**Simple Summary:**

The moor frog *Rana arvalis* can tolerate freezing to low temperatures, up to −16 °C. We performed metabolomic analysis of the liver and hindlimb muscles of frozen and control *R. arvalis*. We found that the moor frog synthesizes glucose and glycerol in similar concentrations as low molecular weight cryoprotectants. This is the first such case reported for the genus *Rana*, which was believed to use glucose only. We found that freezing upregulates glycolysis, with the accumulation of several end products: lactate, alanine, ethanol, and, possibly, 2,3-butanediol. To our knowledge, this is also the first report of ethanol as an end product of glycolysis in terrestrial vertebrates. We observed highly increased concentrations of nucleotide degradation products, implying high level of stress. We found almost no signs of adaptations to reoxygenation stress, with overall low levels of antioxidants. We also performed metabolomics analysis of subcutaneous ice that was found to contain glucose, glycerol, and several other substances.

**Abstract:**

The moor frog *Rana arvalis* is one of a few amphibians that can tolerate freezing to low temperatures, up to −16 °C. In this study, we performed metabolomic analysis of the liver and hindlimb muscles of frozen and control *R. arvalis*. We found that the moor frog synthesizes glucose and glycerol in similar concentrations as low molecular weight cryoprotectants. This is the first such case reported for the genus Rana, which was believed to use glucose only. We found that freezing upregulates glycolysis, with the accumulation of several end products: lactate, alanine, ethanol, and, possibly, 2,3-butanediol. To our knowledge, this is also the first report of ethanol as an end product of glycolysis in terrestrial vertebrates. We observed highly increased concentrations of nucleotide degradation products, implying high level of stress. The Krebs cycle arrest resulted in high concentrations of succinate, which is common for animals. However, we found almost no signs of adaptations to reoxygenation stress, with overall low levels of antioxidants. We also performed metabolomics analysis of subcutaneous ice that was found to contain glucose, glycerol, and several other substances.

## 1. Introduction

Most amphibians live in the warm regions of the planet. However, a few species managed to adapt to cold winters of the North, either by overwintering in water or deep in soil, or by gaining the ability to survive freezing of a part of body water. Studies on the adaptations of amphibians to freezing were started by Schmid [1] on *Rana sylvatica* and two hylids, *Hyla versicolor* and *Pseudacris* (*Hyla*) *crucifer*, and by Berman et al. [2] on the Siberian salamander *Salamandrella keyserlingii*, followed by a multitude of studies (reviewed in [3,4,5,6,7,8]).

Freezing induces severe dramatic changes in body functioning: arrest of breathing and blood circulation, formation of ice inside the body, and severe cell dehydration [9,10]. Freeze-tolerant animals demonstrate a set of responses including hypometabolism, activation of antioxidant defenses, and multiple changes in various regulation pathways [7,11]. Many substances are accumulated in large quantities, including high and low molecular weight cryoprotectants, chaperones, ice-binding, and ice-nucleating proteins to cope with ice formation [11]. Although the responses to freezing in various taxa were formed on a similar biochemical basis, they arose from different starting points and employ a variety of molecules.

In the genus *Rana*, there are two known species tolerant to prolonged freezing (over several weeks). These species overwinter near the soil surface under leaf litter or moss, in tussocks and other substrates that protect animals from the elements. The North American *R. sylvatica* can tolerate up to −16–−18 °C, and maybe even down to −22 °C [8,12]. This species is a well-established model of amphibian freeze tolerance with numerous studies [3,8]. Much less is known on the Eurasian *R. arvalis*. Different populations of this species are capable of surviving freezing at −4–−16 °C [13,14]. Voituron et al. [13] showed that *R. arvalis* synthesized glucose as a cryoprotectant. However, it was present in the liver but not in the muscle and its concentrations were significantly lower than in other freeze-tolerant frog species, which surmised the existence of other cryoprotectants. Oxygen consumption in supercooled state and during freezing was strongly reduced but still active and lactate levels were increased in the liver but not in the muscle [13].

In this study we used 1H-NMR to study the metabolomic response of *R. arvalis* to freezing. We aimed to answer the questions posed by Voituron et al. [13]: if glucose is the only low molecular weight cryoprotectant in *R. arvalis*, to what extent does freezing activate anaerobic metabolism and what end products are formed? We also hoped to glean other insights into the mechanism of response to freezing-related ischemia.

## 2. Materials and Methods

### 2.1. Animals

Specimens of the moor frog were sampled near Chernogolovka town (Moscow oblast, Russia) in end September–early October 2020 during their migration to overwintering sites (N 56.03 E38.40). Earlier studies suggest that animals in these populations can survive freezing to −12 °C [14].

Frogs were placed into a 1 L ventilated plastic container (five animals per container). The containers were filled with wet moss at about 80% humidity. The moor frogs were not fed during the experiment because they do not feed after the migration to overwintering sites in nature. We took 10 animals that were randomly distributed between the control and experimental samples so that both the experimental and control samples contained four males and one female. Temperature was gradually decreased to that observed in natural overwintering sites [14]. The control group was further incubated at 1 °C throughout the experiment. Incubation temperature of the exposed group was gradually decreased to −5 °C (Table 1). Different individuals of *R. arvalis* undergo freezing at temperatures ranging from −1.9 to −3.3 °C [14], so at −5 °C all the animals are frozen. Survival rate at this temperature is 100% [14]. At temperatures above 5 °C, the animals were incubated in TSO-1/80 SPU thermostats (Smolensk SKTB SPU, Smolensk, Russia); from 5 to −5 °C, in a WT-64/75 climatic test chamber (Weiss Umwelttechnik GmbH, Stuttgart, Germany) with the adjustment rate of 0.05 °C/h [15] (Table 1). After 10 days of incubation at −5 °C, frogs of the experimental group were slaughtered without anesthesia; the organs were resected within 20–30 s and frozen in liquid nitrogen.

### 2.2. NMR Measurements

NMR spectra were obtained for protein- and lipid-free extracts of tissue samples of the frozen and control animals. Each sample was weighed prior to homogenization, the typical weight was about 100 mg. The sample was placed in a glass vial and homogenized with a TissueRuptor II homogenizer (Qiagen, Venlo, The Netherlands) in 1600 µL of cold (−20 °C) MeOH, and then 800 µL of water and 1600 µL of cold chloroform were added. The mixture was shaken well in a shaker for 20 min and left at −20 °C for 30 min. Then, the mixture was centrifuged at 16,100× *g*, at 4 °C for 30 min, yielding two immiscible liquid layers separated by a protein layer. The upper aqueous layer (MeOH-H_2_O) was collected and lyophilized. The final set contained five samples for both control and frozen groups. Water was purified to 18.2 MOhm, prepared using the Ultra Clear UV plus water system (SG water, Guenzburg, Germany). Chloroform and methanol were from Panreac (Castellar del Vallès, Spain); D_2_O 99.9% was purchased from Armar Chemicals (Döttingen, Switzerland); all other chemicals were from Sigma-Aldrich (Burlington, MA, USA).

The 1H-NMR procedures were performed in the «Mass spectrometric investigations» SB RAS Center of Collective Use on an AVANCE III HD 700 MHz NMR spectrometer (Bruker BioSpin, Rheinstetten, Germany) with a 16.44 Tesla Ascend cryomagnet [16]. Extracts for NMR measurements were re-dissolved in 600 μL of D_2_O containing 20 mM deuterated phosphate buffer (pH 7.2). In total, 20 μM DSS (sodium 4,4-dimethyl-4-silapentane-1-sulfonic acid) was used as the internal standard. For each sample, we obtained the proton NMR spectra with 64 accumulations. Throughout the measurements, sample temperature was maintained at 25 °C, the detection pulse was 90 degrees, and the repetition time between scans was 12 s. Prior to acquisition, we presaturated the water signal by application of the low power radiation at the water resonance frequency.

Concentrations of metabolites were determined according to the peak area integration relative to DSS. For most of the substances, signal identification was completed according to their published NMR spectra [17] and our proprietary database [18]. In questionable cases we confirmed the identification by checking against commercial standard compounds.

MetaboAnalyst 5.0 web-platform (www.metaboanalyst.ca; accessed date: 10 February 2022) [19] was used for chemometric analysis. Auto data scaling was employed to visualize volcano and loading plots, as well as PCA scores [19]. The Mann–Whitney test was used to check for statistically significant differences between the control and experimental groups.

## 3. Results

### 3.1. NMR Analysis

**Figure 1:**
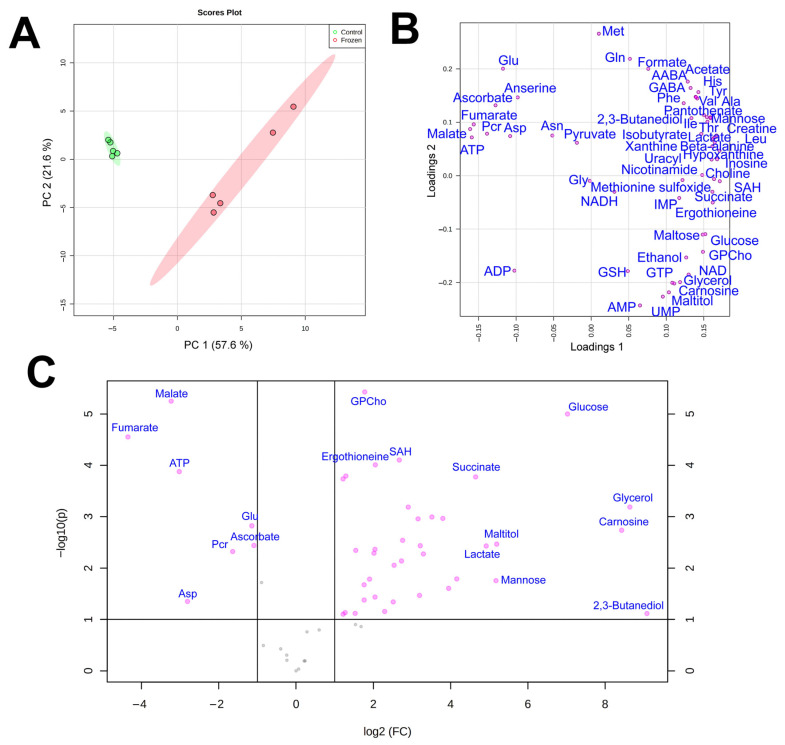
Score (**A**) and loading (**B**) plots of the principal component analysis (PCA) of metabolomic profiles of frozen (red) and control (green) liver samples of *R. arvalis*. The data are auto scaled. Colored ovals indicate 95% confidence regions. (**C**) Volcano plot for frozen and control liver samples. Dots indicate individual substances.

**Figure 2:**
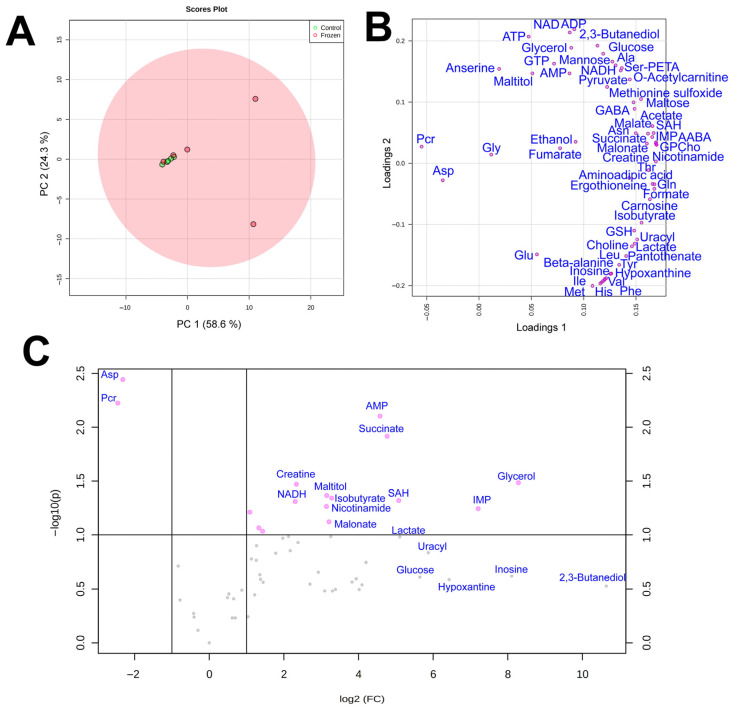
Score (**A**) and loading (**B**) plots of the principal component analysis (PCA) of metabolomic profiles of frozen (red) and control (green) hindlimb muscle samples of *R. arvalis*. The data are auto scaled. (**C**) Volcano plot for frozen and control muscle samples. Dots indicate individual substances.

By using quantitative NMR-based metabolomic profiling we identified 56 metabolites in the liver and 57 in the muscles (Table 2). PCA analysis (Figure 1a) demonstrated that metabolomic profiles of the two liver samples were highly distinct, with little variation in the control and significant in the frozen sample. In the muscles, the plots for the two groups significantly overlapped, with three frozen specimens within or near the control group (Figure 2a).

In PCA loading plots (Figure 1b and Figure 2b), the majority of metabolites were concentrated at the right sides of the plots, indicating a general increase in metabolite concentrations in frozen samples. Volcano plots (Figure 1c and Figure 2c) demonstrated that a statistically significant (*p* < 0.05, fold change > 2) increase was found for 32 metabolites in the liver and for 8 compounds in the muscle, while the decrease was observed for 7 compounds in the liver and only 2 compounds in the muscle. We should note that for a number of metabolites in the muscle the concentration increase was high, but due to very strong sample-to-sample data scattering it was not statistically significant. The highest increase in concentration in both liver and muscle was observed for glycerol, glucose, and 2,3-butanediol, and the decrease for phosphocreatine and aspartate. 

### 3.2. Cryoprotectants

**Figure 3:**
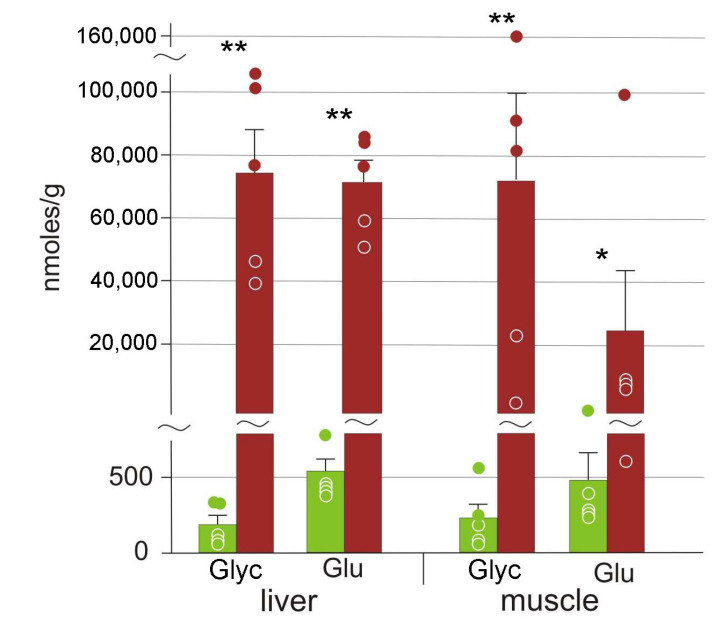
The concentrations of compounds with cryoprotectant properties in *R. arvalis* organs: Glyc stands for glycerol, and Glu, for glucose. Green columns, control; red, frozen; bar, SE; circles, individual data points; * Mann–Whitney test *p* < 0.05; ** *p* < 0.01.

We detected two cryoprotective substances, glucose and glycerol, that were present in frozen organs in very high quantities (Figure 3). Average concentrations of these cryoprotectants increased in frozen samples by 50–400-fold. The increase was less pronounced in the muscle with significantly higher variation: values ranged from very high to similar to those in the control samples. Average concentrations of another three potential cryoprotectants, mannose, maltose, and maltitol, also increased (Table 2).

### 3.3. Energy Metabolism

**Figure 4:**
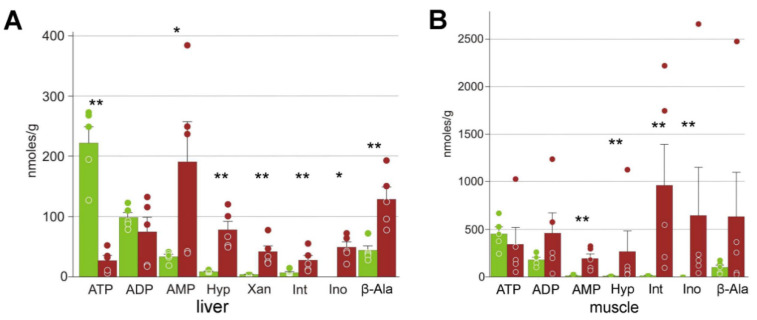
Products of nucleotide degradation in *R. arvalis* organs: Hyp, hypoxanthine; Int, inosinate; Ino, inosine; β-Ala, β-alanine. (**A**) Liver; (**B**) hindlimb muscle. Green columns, control; red, frozen; bar, SE; circles, individual data points; * Mann–Whitney test *p* < 0.05; ** *p* < 0.01.

In the liver, we found the expected depleted energy molecules, with on average 8-fold lower ATP and 5-fold higher AMP concentrations (Figure 4). In the muscles only the AMP increase was statistically significant. We found a dramatic increase in the purine degradation pathway intermediates: xanthine, hypoxanthine, inosine, and inosinate in the liver, and of the latter three in the muscles. The pyrimidine degradation intermediates, β-aminoisobutyrate and β-alanine, also had higher average concentrations in the frozen organs, but the differences were statistically significant only for β-alanine in the liver (Figure 4, Table 2).

**Figure 5:**
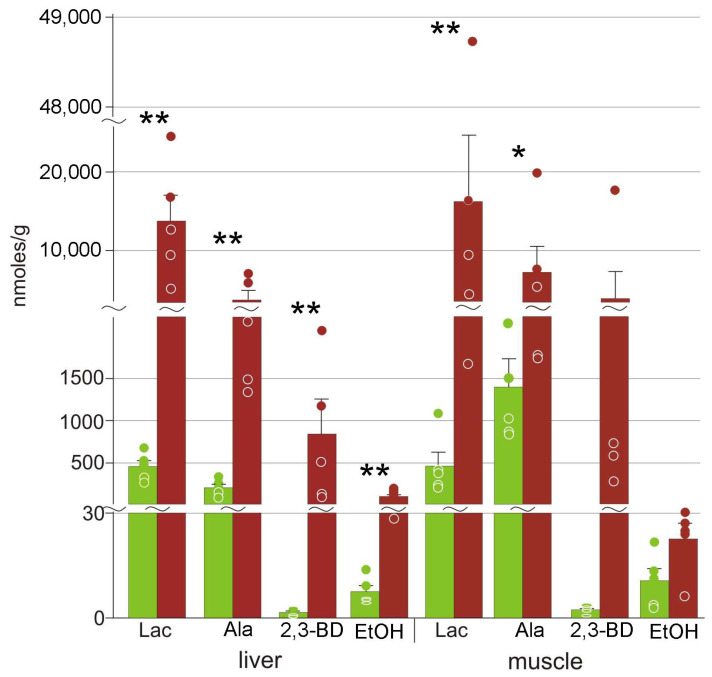
Concentrations of glycolysis end products in *R. arvalis* organs: Lac, lactate; Ala, alanine; 2,3-BD, 2,3-butanediol; EtOH, ethanol. Green columns, control; red, frozen; bar, SE; circles, individual data points; * Mann–Whitney test *p* < 0.05; ** *p* < 0.01.

In both muscle and liver, we detected small amounts of pyruvate (Table 2). The quantities of lactate were significantly increased in both liver and muscles (Figure 5). Alanine was also present in increased concentrations, although lower than those of lactate. We also detected elevated concentrations of ethanol and 2,3-butanediol in the liver of frozen frogs (Figure 5). The concentrations of phosphocreatine decreased, and those of creatine increased relative to the control group (Table 2).

**Figure 6:**
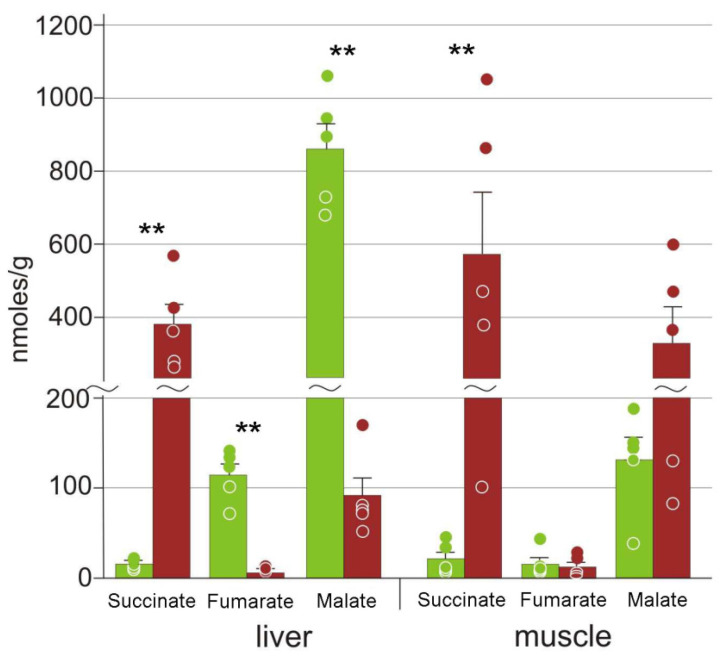
Concentrations of Krebs cycle intermediates in the *R. arvalis* organs. Green columns, control; red, frozen; bar, SE; circles, individual data points; ** *p* < 0.01.

Three components of the Krebs cycle were found in the liver: succinate, fumarate, and malate; only the former two were detected in the muscles. The concentrations of succinate increased significantly in both tissues, while those of fumarate and malate were decreased in the liver (Figure 6).

### 3.4. Amino Acids

**Figure 7:**
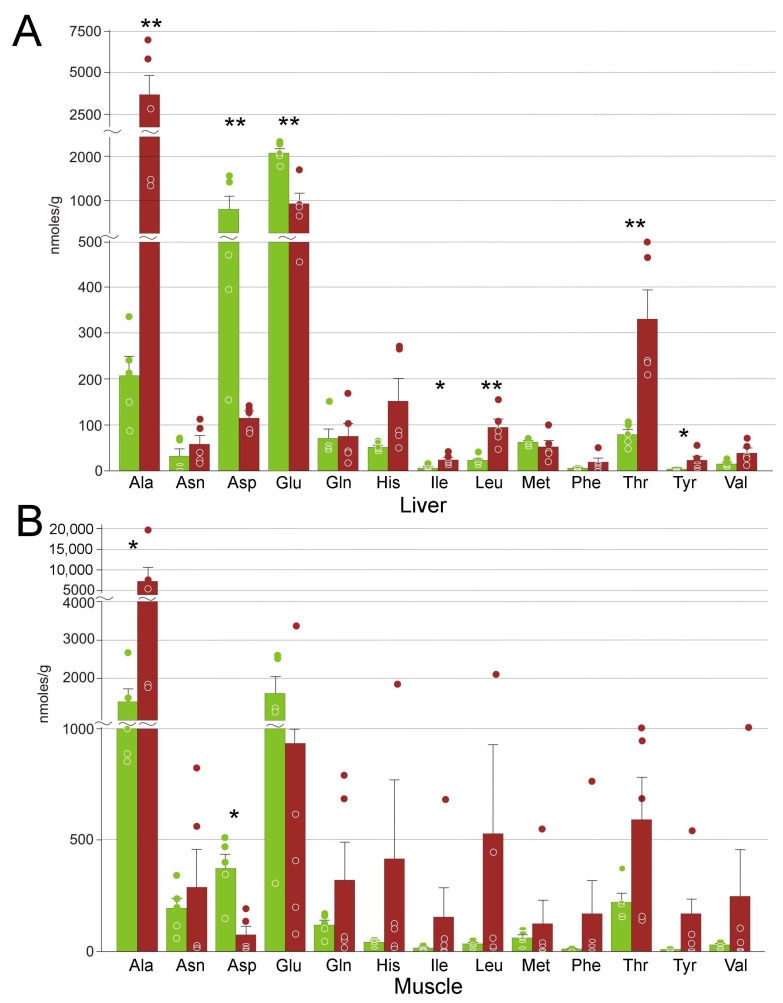
Concentrations of free amino acids in *R. arvalis* organs. (**A**) Liver; (**B**) muscle. Green columns, control; red, frozen; bar, SE; circles, individual data points; * Mann–Whitney test *p* < 0.05; ** *p* < 0.01.

A total of 13 proteinogenic amino acids were detected in both tissues. In the liver, concentrations of seven of them changed significantly: for five, we observed an increase, and for glutamate and aspartate a decrease (Figure 7). In the muscle, average changes in the concentrations of the free amino acids were dramatic, but with very high variation, so only for two of them, alanine and aspartate, these changes were statistically significant. We should also note significant changes in amino acid derivatives, ergothioneine, carnosine, methionine sulfoxide, and S-adenosylhomocysteine in the liver, and of the latter in the muscle (Table 2).

### 3.5. Subcutaneous Ice

We determined the composition of a piece of subcutaneous ice located near the hindlimb muscle for a single specimen of *R. arvalis*. We found it to contain significant concentrations of certain compounds (Table 3). The concentrations of glucose and glycerol were several fold lower than in frozen organs but much higher than in control ones (Table 2). Subcutaneous ice also contained glycolysis end products, creatine, glycerophosphocholine, as well as minor amounts of other substances.

## 4. Discussion

This study was the first attempt to examine the freezing response of *R. arvalis* using metabolomics. It has certain limitations, mainly the fact that we took only two samples (control and frozen frogs) at two temperature points, while it would be much better to explore different freezing temperatures, as well as thawed frogs. Freezing results in tissue dehydration, which presents certain problems at results interpretation, as discussed in [20]. However, we believe we can make certain inferences from our data.

### 4.1. Cryoprotectants in the Moor Frog

Several frog species are known to be tolerant to freezing: the ranids *Rana sylvatica* [1,6] and *R. arvalis* [13,14], as well as several hylids (*Hyla japonica*, *H. versicolor*, and *H. chrysocelis*) [1,21,22,23] and *Pseudacris* (*Hyla*) *crucifer*, *P. maculata*, and *P. triseriata* [1,24]. These species can tolerate freezing to temperatures as low as −35 °C for *H. japonica* [21], −18 °C for *R. sylvatica* [12], and −16 °C for *R. arvalis* [14]. This remarkable freezing tolerance is mediated by multiple mechanisms [3,7]. These include the accumulation of high molecular weight (e.g., antifreeze proteins [25,26,27]) and low molecular weight molecules [28]. In this study we focused on the latter; there are a number of such substances employed by different animals [28], but in the amphibians these are represented by glycerol, glucose, and urea.

It is believed that amphibian species use mostly either glucose or glycerol: frogs of the genera *Rana* and *Pseudacris* use glucose, those of the genus *Hyla* rely mostly on glycerol [23,29,30,31], while *S. keyserlingii* uses glycerol only [2,20]. *R. sylvatica* was demonstrated to accumulate glucose but not glycerol [15,32,33], and the same was shown for *P. maculata* [34]. Hylids are believed to synthesize more glycerol than glucose [23,35,36], but there are exceptions to this rule [37]. Based on this, one could imply that different cryoprotectants are synthesized in different frog genera [29].

There were two studies on *R. arvalis* freezing biochemistry that were performed on populations from Denmark and Siberia [13,38]. Voituron et al. [13] found that glucose acted as a cryoprotectant, but they did not assay the amount of glycerol. In this study we detected very high concentrations of glycerol in both liver and muscles of the moor frog, on average 70 μmoles/g in both tissues (Figure 3). This is approximately similar to the amount of the accumulated glucose. Thus, we found out that *R. arvalis* employs both glucose and glycerol as cryoprotectants. Bulakhova and Shishikina [38] estimated glycogen reserves in liver and muscle before the winter and demonstrated a high degree of glycogen degradation upon freezing.

We also found statistically significant increases in maltose, mannose, and maltitol in the liver (Table 2). In the muscles, the average values for these compounds also increased, but these increases were not statistically significant, except for maltitol. Their concentrations were not high compared to glucose and glycerol, up to 800 nmoles/g, so these molecules obviously cannot act as cryoprotectants. We hypothesize that the increase in the amount of these three substances is associated with the upregulation of glycerol and glucose biosynthesis pathways. Maltose, mannose, and maltitol are formed by interconversion of sugars. Thus, the increased amounts of glucose and glycerol provide more substrate for possible side reactions.

### 4.2. Glucose Variation Patterns

Voituron et al. [13] found that glucose concentrations in *R. arvalis* increased only in the liver, up to 40 μmoles/g, while in our study it ranged from 50 to 84 μmoles/g. Glucose concentration in the muscle in [13] was low, 2.5–3.5 μmoles/g in frozen individuals vs. up to 100 μmoles/g in our study. These differences between the two studies are in agreement with lower cold tolerance detected in [13], −4 °C vs. up to −12 °C in the Moscow population studied by us and −16 °C in a West Siberian population [14]. Higher concentrations of glucose in our data compared to [13] might be due to the differences between the populations. Differences in cold tolerance and in biochemistry between geographically distant populations are well established in *R. sylvatica* [8,12,15,39], so it would be reasonable to expect the same for *R. arvalis*. Although the Danish and Moscow populations were demonstrated to be genetically similar [14], there still might be significant adaptations to local environments: maritime climate in Denmark vs. continental in Moscow. On the other hand, differences in experimental protocols, freezing modes, immobilization, and inoculation methods (discussed at length in [14]), also probably contributed, so the observed discrepancies between the two studies are probably due to the combination of all factors.

Another observed phenomenon was high variation in glucose and glycerol, as well as many other compounds, in the muscles. Glycerol concentration varied from 2757 to 160,410 nmoles/g, and glucose 607 to 99,500 nmoles/g, i.e., from background to very high values (Table 2). The quantity of the accumulated cryoprotectants obviously depends on the available glycogen deposits, and presumably also on age, sex, genetic background, and general physical condition. In addition, the extremities are the first to freeze [40], so they have less time for metabolic adaptation. According to our observations [38], muscles of the moor frog in the fall contain four times less glycogen compared to the liver, and so depend on energy influx from the body. This was also demonstrated for other freeze-tolerant amphibians: individual organs have different ratios of glycolysis end products and demonstrate organ-specific metabolic rates [10]. Thus, the content of various molecules in the muscles, also including glycolysis end products, may represent a ‘snapshot’ of current metabolic conditions, including physical activity.

### 4.3. Energetic Processes during Freezing

Freezing leads to blood flow arrest and ischemia, and the highly reduced oxygen levels inhibit oxidative phosphorylation. This results in dramatic energy deficit; the main ways to compensate for this are to reduce metabolic rate and to upregulate glycolysis [7,41]. Voituron et al. [13] demonstrated increased concentrations of lactate in the liver but not in the muscle of the frozen *R. arvalis*. In this study, we detected significantly higher lactate content both in the liver and the hindlimb muscle of the moor frog (Figure 5). However, lactate is not the only end product: in liver, we detected elevated concentrations of alanine, ethanol, and 2,3-butanediol (Figure 5). Alanine is an alternative end product of glycolysis in various organisms including amphibians, e.g., *R. sylvatica* [42,43] and *P. crucifer* [44]. Alanine was found in lower quantities compared to lactate (Figure 5).

Ethanol is also a well-known end product of glycolysis. However, in vertebrates it appears to be very limited, reported in this role only in hypoxia-tolerant fish [41,45,46]. In the moor frog, ethanol concentrations increased about 14-fold in the liver, with the final concentrations two orders of magnitude lower than those of lactate (Figure 5, Table 2). We suggest that the formation of ethanol is the result of minor activity of one of the pyruvate conversion pathways. Therefore, this is, to our knowledge, the first case of ethanol formation in glycolysis in terrestrial vertebrates.

We detected significant quantities of 2,3-butanediol (Figure 5, Table 2). Although higher absolute concentrations were found in frozen muscles, the differences were not statistically significant due to high variation (Section 4.2). Relatively little is known on the role of 2,3-butanediol in animals. This substance was found in high quantities in the Siberian wood frog exposed to anoxia [47], which led to the hypothesis that it is an alternative end product of glycolysis. However, this hypothesis still has to be verified.

It is known that hypoxia/ischemia results in the arrest of the Krebs cycle with the accumulation of succinate in vertebrates [48]. This was observed for frozen *R. sylvatica* [33], as well as for the Siberian wood frog *R. amurensis* and the red-eared slider turtle *Trachemys scripta* under anoxia [47,49]. In this study, we found a dramatic (25-fold) increase in succinate concentrations in liver and muscles of the moor frog (Figure 6). Voituron et al. [13] demonstrated that in *R. arvalis*, oxygen consumption persisted but reduced 10–16-fold after a temperature decrease from 4 °C to −2–−4 °C. The observed succinate accumulation suggests a substantial metabolism shift towards anaerobic glycolysis.

We should also note that average concentrations of malonate increase almost 10-fold in frozen muscles (Table 2). Malonate is well-known as an inhibitor of succinate dehydrogenase that is used to reduce reoxygenation injury [48,50,51]. Studies on the natural synthesis of malonate in vertebrates and its function are rarer [52,53,54]. It would be tantalizing to suggest that the moor frog synthesizes malonate in order to counteract reoxygenation stress. However, in this case it would be hard to account for the fact that malonate was found in muscles but not in the liver (Table 2), while it would be more appropriate to use it to protect the more important internal organ. This issue obviously requires further study.

### 4.4. Markers of Stress

Transition from oxidative phosphorylation to glycolysis results in a significant energy deficit due to much lower efficiency of anaerobic glycolysis, which can be partially compensated by reducing the metabolic rate [7]. In the liver, we found signs of energetic stress manifested by significantly lower ATP and higher AMP concentrations. In muscles, however, only the increase in AMP concentrations was statistically significant. We also detected a significant decrease in phosphocreatine concentrations with a concomitant increase in creatine (Table 2).

We also observed a profound increase in the concentrations of xanthine, hypoxanthine, β-alanine, and β-aminoisobutyrate (Figure 4, Table 2), which are nucleotide degradation products and are often observed in decaying tissues [55,56,57,58]. This indicates that the stress is so dramatic that it results in nucleotide degradation. It is noteworthy that nucleotide degradation products were found in the frozen Siberian salamander but not in the Siberian wood frog under anoxia using the same methods [20,47], suggesting that hypoxia is less damaging compared to freezing.

It is well known that freezing-related ischemia with subsequent reoxygenation results in oxidative stress [41]. This may be manifested in increased amounts of antioxidants in frozen animals, the so-called Preparation for Oxidative Stress [59,60]. This is observed, e.g., in turtles [61,62], snakes [63], and lizards [64]. However, for *R. sylvatica* it was shown that antioxidant systems are upregulated not in all tissues [65,66]. Specifically, increased glutathione concentrations were observed only in frozen brain and kidneys in this species [65]. We observed only a slight increase in GSH in liver and none in the muscles. We should also note that GSH concentrations were much lower compared to *R. sylvatica*, on the average 2-fold in muscles and 8-fold in liver. Ascorbate, carnosine, and ergothioneine also have antioxidant activities. Concentrations of these molecules did not demonstrate any changes in the muscle. In liver, ascorbate and carnosine content was significantly lower, and of ergothioneine, higher. These data suggest a mixed response to oxidative stress in *R. arvalis*, similar to that observed in *R. sylvatica* [65].

### 4.5. Subcutaneous Ice

We performed only a single measurement of the composition of subcutaneous ice and thus cannot make any inferences about the range of concentrations of individual substances. However, it is obvious that the ice is composed not just of pure water but contains significant concentrations of some compounds (Table 3). The concentrations of glucose and glycerol are several fold lower than in frozen organs but much higher than in control ones (Table 2). Subcutaneous ice also contains glycolysis end products, creatine, glycerophosphocholine, etc. On the one hand, most of these substances also have the highest concentration in the muscles, so it could be said that ice composition simply reflects metabolite content in the tissues. On the other hand, some of the compounds ubiquitous in the muscles were not found in the ice while less abundant ones were. Of all amino acids, only glutamine was found in the ice, while amino acids with higher concentrations in tissues were absent.

Subcutaneous liquid in amphibians is a part of the lymphatic system. During freezing, lymph is mixed with water exiting the cells. Subcutaneous ice forms before the organs are frozen, so its composition must reflect the early stages of freezing. Currently, we cannot say whether the substances in subcutaneous ice have any adaptive value; if we assume this hypothesis, we can suggest that glycerol and glucose might have a role in the regulation of the freezing point of water. The temperature at which ice nucleation begins has a significant impact on survival rate [67], and cryoprotectant content affects the freezing point of the solute. Glutamine in extracellular ice could be the result of ammonia transport between organs. The presence of succinate might be a way to reduce its intracellular concentrations, decreasing reoxygenation stress. However, all these assumptions are currently speculative and have to be reinforced by more data.

## 5. Conclusions

In this study we made several major observations on the freezing response of the moor frog *R. arvalis*. This species turned out to employ similar levels of glucose and glycerol as cryoprotectants. This is the first reported case of glycerol as a cryoprotectant in Ranidae. Glycolysis in *R. arvalis* results in accumulation of several end products: lactate, alanine, ethanol, and presumably 2.3-butanediol. For ethanol, it appears to be the first case for terrestrial vertebrates. We also gained insights into the metabolome composition of frogs and into freezing response in particular.

## Figures and Tables

**Table 1:** Freezing protocol for the moor frog individuals.

T, °C	Duration, Days
5	30
1	15
−1	20
−2	2
−3	2
−5	10

**Table 2:** Average concentrations of metabolites in the organs of *R. arvalis* (nmoles per gram of wet tissue) and standard error (n = 5); n/a, not detected. Statistical significance between frozen and control samples: * Mann–Whitney test *p* < 0.05; ** *p* < 0.01.

	Liver		Muscle	
Compound	Control	Frozen	Control	Frozen
2-aminoadipate	n/a	n/a	126 ± 21	180 ± 50
2,3-butanediol	2.4 ± 0.4	800 ± 400 **	1.6 ± 0.4	4000 ± 3000
Acetate	1000 ± 190	1500 ± 300	1110 ± 130	2700 ± 1000
ADP	99 ± 18	75 ± 24	179 ± 27	460 ± 210
Alanine	210 ± 90	3700 ± 1100 **	1400 ± 300	7000 ± 3000 *
α-aminobutyrate	2.9 ± 0.8	8 ± 3	6.8 ± 0.9	18 ± 9
AMP	33 ± 9	190 ± 70	8.0 ± 1.0	190 ± 50 **
Anserine	550 ± 140	300 ± 60	3700 ± 700	2100 ± 900
Ascorbate	82 ± 21	38 ± 5 **	n/a	n/a
Asparagine	60 ± 40	32 ± 16	190 ± 50	290 ± 170
Aspartate	800 ± 600	114 ± 11 **	370 ± 60	80 ± 40 *
ATP	220 ± 60	27 ± 8	450 ± 70	340 ± 180
β-alanine	44 ± 17	129 ± 20 **	97 ± 26	600 ± 500
β-aminoisobutyrate	0.5 ± 0.4	3.1 ± 1.3	6.3 ± 0.9	28 ± 14
Carnosine	1.2 ± 0.3	420 ± 90 *	3500 ± 600	8400 ± 2800
Choline	12 ± 4	87 ± 14 **	11.7 ± 2.0	220 ± 140
Creatine	35 ± 8	540 ± 180 **	4700 ± 600	24,000 ± 7000 *
Ergothioneine	32 ± 10	131 ± 13 **	14 ± 3	61 ± 25
Ethanol	8 ± 4	106 ± 20 **	11 ± 3	23 ± 4
Formate	46 ± 12	54 ± 16	52 ± 7	113 ± 40
Fumarate	115 ± 28	6.0 ± 2.0 **	15 ± 7	12 ± 5
GABA	21 ± 7	48 ± 13	8 ± 4	29 ± 12
Glucose	540 ± 180	71,000 ± 7000 **	480 ± 190	24,000 ± 12,000 **
Glutamate	2090 ± 220	950 ± 220 **	1600 ± 400	900 ± 600
Glutamine	70 ± 50	75 ± 27	118 ± 21	320 ± 170
Glycerol	190 ± 130	74,000 ± 14,000 **	200 ± 90	72,000 ± 28,000 **
Glycerophosphocholine	1820 ± 150	6200 ± 400 **	1090 ± 230	4300 ± 1700
Glycine	34 ± 9	n/a	360 ± 40	n/a
GSH	170 ± 40	204 ± 18 **	96 ± 15	150 ± 60
GTP	3.6 ± 0.5	24 ± 5 **	23 ± 4	17 ± 8
Histidine	53 ± 9	150 ± 50	40 ± 7	400 ± 300
Hypoxanthine	8.8 ± 0.9	78 ± 14	3.1 ± 0.5	270 ± 220 **
Inosinate	7 ± 4	28 ± 8	7.4 ± 1.8	1000 ± 400 **
Inosine	n/a	49 ± 9	1 ± 1	643 ± 506 **
Isobutyrate	1.9 ± 0.4	6.9 ± 1.8 **	0.9 ± 0.3	9 ± 3 **
Isoleucine	7 ± 5	25 ± 6 *	15 ± 4	150 ± 130
Lactate	460 ± 160	14,000 ± 3000 **	470 ± 160	16,000 ± 9000 **
Leucine	23 ± 11	95 ± 18 **	34 ± 8	500 ± 400
Malate	860 ± 160	92 ± 20 **	131 ± 25	300 ± 100
Malonate	n/a	n/a	38 ± 5	360 ± 160 **
Maltitol	3.1 ± 1.1	116 ± 28 **	n/a	8 ± 3 **
Maltose	107 ± 12	250 ± 21 **	104 ± 18	240 ± 140
Mannose	6 ± 3	220 ± 70 **	52 ± 24	900 ± 700
Methionine	63 ± 7	53 ± 13	60 ± 15	120 ± 110
Methionine sulfoxide	16.0 ± 0.9	54 ± 16 *	2.2 ± 0.3	10 ± 5
NAD	31 ± 7	76 ± 6	86 ± 13	120 ± 40
NADH	3.8 ± 1.0	4.4 ± 0.9	4.1 ± 0.7	20 ± 7 **
Nicotinamide	4 ± 4	25 ± 6	14 ± 4	130 ± 50 *
O-Acetylcarnitine	n/a	n/a	8.9 ± 2.9	80 ± 40 **
Pantothenate	0.5 ± 0.3	5.3 ± 1.8	1.9 ± 0.4	15 ± 10
Phenylalanine	6.2 ± 0.7	20 ± 8	10 ± 3	170 ± 150
Phosphocreatine	27 ± 6	9 ± 4 *	3200 ± 700	590 ± 190 **
Pyruvate	2.0 ± 0.3	1.7 ± 0.4	9 ± 5	14 ± 9
S-adenosylhomocysteine	n/a	24 ± 3 **	n/a	26 ± 11 **
Serine-phosphoethanolamine	n/a	n/a	1250 ± 260	2300 ± 1000
Succinate	15 ± 6	380 ± 60 **	21 ± 8	570 ± 170 **
Threonine	80 ± 23	330 ± 60 **	220 ± 40	590 ± 190
Tyrosine	4.8 ± 0.7	24 ± 9 *	9 ± 3	130 ± 100
UMP	8.8 ± 1.0	59 ± 14 **	n/a	n/a
Uracil	n/a	24 ± 5 **	n/a	70 ± 40 **
Valine	16 ± 8	39 ± 10	29 ± 6	250 ± 210
Xanthine	4.3 ± 0.6	42 ± 10 **	n/a	n/a

**Table 3:** Compounds found in a piece of subcutaneous ice at the hindlimb of the frozen *R. arvalis*.

Compound	Concentration, nM
Glycerol	18,000
Glucose	8000
Lactate	1400
Creatine	260
Alanine	160
Glycerophosphocholine	160
2,3-Butanediol	62
Succinate	57
Carnosine	52
Acetate	51
Glutamine	24
Creatine phosphate	18
Pyruvate	2.9

## Data Availability

Not applicable.
